# Unexpected sudden death in pregnancy – arrhythmogenic right ventricular cardiomyopathy/dysplasia: a case report

**DOI:** 10.1080/20961790.2017.1325548

**Published:** 2017-05-23

**Authors:** Amal Nishantha Vadysinghe, Rankothge Pemasiri Jayasooriya, G. Keerthi Kumara Gunatilake, Murugupillai Sivasubramanium

**Affiliations:** a Department of Forensic Medicine, University of Peradeniya, Peradeniya, Sri Lanka; b Forensic Unit, General Hospital Gampaha, Gampaha, Sri Lanka; c Forensic Unit, Teaching Hospital Kandy, Kandy, Sri Lanka

**Keywords:** Forensic science, sudden death, pregnancy, arrhythmogenic right ventricular cardiomyopathy/ dysplasia (ARVC/D)

## Abstract

Cardiovascular disease is an important contributor to maternal mortality in both developing and developed countries. Systematic search for cardiac disease is usually not performed during pregnancy despite hypertensive disease, undiagnosed pulmonary hypertension and cardiomyopathies being recognized as major health problems in these settings. This article reported a 27-year-old female who was normal on clinical examination and basic investigations, and on an antenatal visit was found collapsed in the toilet of her house and was pronounced dead on admission to hospital. She was found to be in the 11th week of pregnancy and had no history of significant illness in the past. Autopsy did not reveal any obvious macroscopic pathology except for a significant amount of epicardial fat infiltrating into myocardium of right ventricle. Detailed histopathological examination of the heart demonstrated fibro-fatty replacement of the heart muscle. The cause of death was arrhythmogenic right ventricular cardiomyopathy/dysplasia (ARVC/D). ARVC/D can cause unexpected sudden death during pregnancy. Therefore, it is recommended that an ECG and echocardiogram be included as screening tests during antenatal follow-up to minimize preventable cardiac deaths like ARVC/D.

## Introduction

Heart disease complicating pregnancy is one of the leading causes of maternal deaths in Sri Lanka. While direct causes of maternal deaths such as complications of hypertension, obstetric haemorrhage and sepsis remain the commonest causes of maternal death, cardiovascular disease emerges as an important contributor to maternal mortality in developing countries and the developed countries [1,2]. Systematic search for cardiac disease is usually not performed during pregnancy despite hypertensive disease, undiagnosed pulmonary hypertension and cardiomyopathies being recognized as major health problems in this setting. This article discusses a preventable cardiovascular disease which caused sudden death of a young pregnant mother underscoring the need for cardiovascular screening in pregnancy and the importance of detailed histopathological screening in maternal deaths.

## Case history

A 27-year-old apparently healthy female of Sri Lankan origin, conceived a baby three months after discontinuing the oral contraceptive pill. Her urine was positive for human chorionic gonadotropin on the second month after her last menstruation. Antenatal visit revealed that the clinical examination, full blood count and Urine Full report were normal. Approximately in 11th week of amenorrhea, she was found collapsed in the toilet of her house and was pronounced dead on admission to hospital. She had not complained of shortness of breathing, tightness of chest, syncopal attacks or palpitation in her day-to-day activities. She did not have any significant past medical or surgical histories and engaged in mild/moderate physical activities at home and at her occupation as a teacher. There was no history of sudden unexpected deaths or diagnosed case of arrhythmogenic right ventricular cardiomyopathy/ dysplasia (ARVC/D) of family members. A medico-legal autopsy was requested by the inquirer of sudden death.

Autopsy revealed an Asian Caucasoid female of 158 cm in height and 52 kg in weight. There was no evidence of nutritional deficiencies, congenital abnormalities, disease conditions or trauma. All organs were placed in normal anatomical position. The heart weighed 255 g. The right ventricle appeared moderately dilated with wall thickness of 1–3 mm. The epicardial fat thickness of right ventricle was 4–5 mm and infiltrating into the myocardium (Figure S1(A)). Some areas of the myocardium showed thinning of wall to about 1 mm with no evidence of aneurysm formation. The right atrium, left atrium and left ventricle, all valves and pulmonary and aortic vasculature were macroscopically normal. Normal anatomy was identified in coronary circulation with mild atherosclerotic occlusions only at the proximal one-third of left anterior descending artery. A 5 cm length foetus (13 g) was found intrauterine just below the opening of the right fallopian tube. No other macroscopic abnormalities were detected.

Microbiological investigations and toxicological screening were negative. Histology revealed significant replacement of myocardial tissue of the right ventricle with fibro-fatty tissue in haematoxylin and eosin (H&E) (Figure S1(B)) and Masson's trichrome stains (Figure S1(C)). No inflammatory cells were identified. Right and left atrium, left ventricle, all coronaries and valves were normal. Histopathological screening of other organs revealed no abnormalities. Genetic studies were not done on the deceased but relatives have been advised regarding diagnostic availability for ARVC/D.

The cause of death was given as ARVC/D in first trimester of pregnancy. The circumstance was concluded as natural.

## Discussion

ARVC/D is a rare inherited disease of the cardiac muscle [3] and a leading cause of arrhythmia in the young [4]. The initial presentation itself is sudden death of an athletic young person [2]. The prevalence of ARVC/D is known to be 1:1000 to 1:1250 in the general population [5]. Some studies revealed a prevalence of 1:5000 in the general population and 1:2000 in some European countries [6,7]. It is responsible for approximately 20% of sudden cardiac deaths among those who are less than 35 years of age. However, ARVC/D may be diagnosed at any age and may result in sudden death usually between 15 and 45 years with a slightly male preponderance [8]. It is postulated that this may be due to the influence of male hormones on expression [9] or high intensity of physical activity [10]. It is an autosomal dominant condition which is mostly hereditary and associated with mutation in gene encoding proteins of the intercalated disc [11]. However, most patients with ‘definite’ ARVC phenotype by task force 2010 host mutations in desmosomal genes while weaker ARVC phenotypes host variants/mutations in other DCM genes causing a disease spectrum [12]. Thirteen different chromosomal loci have been identified to be associated with this disease [12–15]. Although chances of inheriting ARVC/D vary, some may inherit the mutation but not develop the condition [2]. Genetic studies are important to confirm the diagnosis of the disease and to take preventing measures among blood relatives after clinical or death investigation. Molecular studies were not done during autopsy but the relatives were informed of the importance of screening for ARVC/D among family members as a preventive measure after confirmation by histology.

In this case, there was no history of sudden deaths among family members. This highlights the importance of molecular autopsies especially in the absence of significant autopsy findings. ARVC/D may be associated with syncope attacks, chest pain, palpitation [16], etc. However, history did not reveal that these were experienced by the deceased. Therefore, ARVC/D can be considered as a cause of sudden unexpected death in pregnancy [17].

According to the symptoms and presentation, it may be determined that this victim was probably in the concealed phase of ARVC/D. The volume overload and hormonal changes during pregnancy may have caused strain to the right ventricle and thereby predisposing to sudden cardiac death following arrhythmic episode [18] as a mechanism of death in this case.

The development of ARVC/D is by two mechanisms; one of the degeneration types of myocytes associated with genes and second is interstitial inflammation either due to infections or autoimmune reaction [19]. The hallmark of the disease is the fatty infiltration replacing cardiac cells which may be associated with fibrosis and infiltration by inflammatory cells. A similar appearance may be seen in the left ventricular wall [20]. In this case, mature fatty and fibrous tissues were confining only to the right ventricle.

Maternal deaths are not usually of medico-legal importance unless the death is associated with mainly criminal abortion, medical negligence [21]. The leading causes of sudden deaths during natal and postnatal period include sudden arrhythmic death syndrome, cardiomyopathies, dissection of major vessels, congenital heart disease and valvular heart diseases [22], or gynaecological causes include abortion, ruptured tubal rupture, etc. Age-related gender discrepancy of cardiovascular diseases were not applied to the case we presented as deceased was 27 years old. However, it is essential to investigate sudden unexpected death in pregnancy comprehensively in order to determine the cause of death and to determine the necessity to screen relatives of the deceased to prevent occurrence of similar fatalities.

There is no single diagnostic clinical test for ARVC/D as changes in cardiac muscles are often minor or build up in a patchy pattern. Furthermore, since many of the changes and symptoms could also be caused by a number of other conditions, ARVC/D should be suspected if symptoms are present or if there is a family history [23].

In ARVC/D, ECG may show a complete/incomplete right bundle branch block with T wave inversion in leads V1–V3, and beyond ventricular, tachycardia, and epsilon wave. Right ventricular image studies may show thinning and dilatation of the right ventricular wall or dysfunction. Biopsy may reveal fibro-fatty infiltration of right ventricle [21].

If these tests were included in the panel of investigations done during routine pregnancy, this death may have been prevented. However, these tests were not done in this victim during life as there is no indication to do so in the system currently existing in Sri Lanka.

Though ARVC/D is a potentially life threatening disease, it may be managed conservatively even during pregnancy or with beta-blocker or sometime with implantable cardioverter-defibrillator who are at high risk, and one study revealed that approximately 13% and 5% were complicated by ventricular arrhythmias and heart failure, respectively [24].

There is no single lab investigation to diagnose clinically ARVC/D and many parameters are subjective. Existing criteria were revisited in 2010 (2010 Task Force Criteria) and it is really helpful to take necessary management in those who are at risk [25].

Close monitoring should be done in women of reproductive age group especially those who have a family history of unexplained sudden deaths and cardiovascular symptoms in early pregnancy with ECG and echocardiography [16]. Also refinement of management protocols and guidelines is mandatory to minimize the risk [26].

## Supplementary Material

Supp_mat_TFSR_Figure_1_1325548.jpg
