# Supplementary figures and images for: Unexpected sudden death in pregnancy – arrhythmogenic right ventricular cardiomyopathy/dysplasia: a case report
Source: Forensic Sci Res. 2017 May 23;2(3):161–3. doi: 10.1080/20961790.2017.1325548 (PMC6197088; doi:10.1080/20961790.2017.1325548)

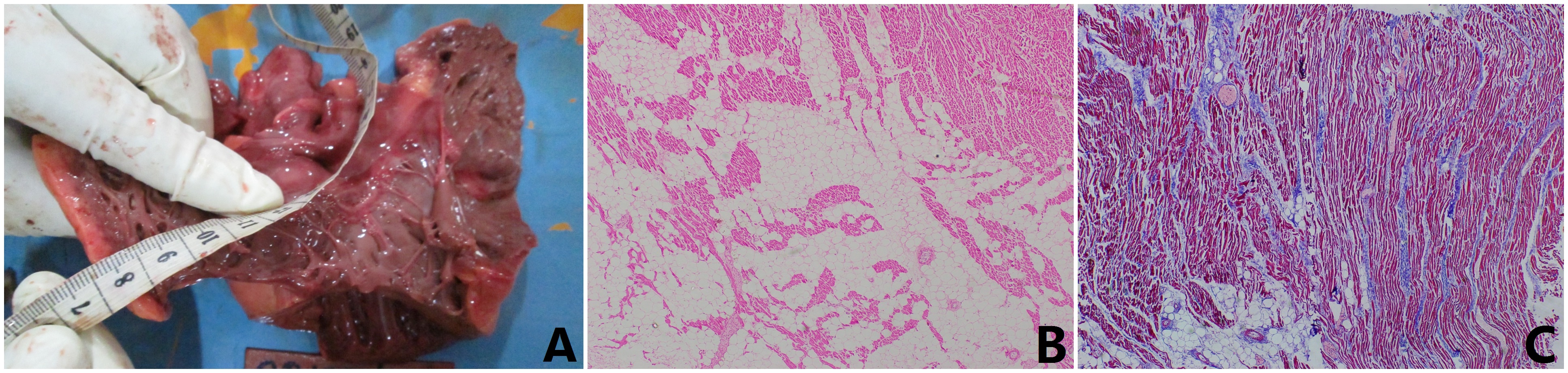

Supplement: Supp_mat_TFSR_Figure_1_1325548.jpg [file TFSR_A_1325548_SM1385.jpg]
